# Stable Mydriasis After Intracameral Injection of a Combination of Mydriatics and Anesthetic During Cataract Surgery: A Real-Life, Multicenter Study

**DOI:** 10.1089/jop.2020.0001

**Published:** 2020-12-11

**Authors:** Dariusz Kęcik, Iwona Grabska-Liberek, Piotr Jurowski, Ewa Mrukwa-Kominek, Wojciech Omulecki, Bożena Romanowska-Dixon, Jacek P. Szaflik, Wanda Romaniuk, Jerzy Szaflik, Dorota Kopacz, Katarzyna Mianowska

**Affiliations:** ^1^ Department of Ophthalmology, Medical University of Warsaw, Warsaw, Poland.; ^2^ Department of Ophthalmology, Medical Centre for Postgraduate Education, Warsaw, Poland.; ^3^ Department of Eye Diseases, Medical University of Lodz, Lodz, Poland.; ^4^ Department of Ophthalmology, School of Medicine in Katowice, Medical University of Silesia, Katowice, Poland.; ^5^ Department of Ophthalmology, Medical University of Lodz, University Hospital No 1, Lodz, Poland.; ^6^ Department of Ophthalmology and Ocular Oncology, Jagiellonian University Medical College, Cracow, Poland.

**Keywords:** cataract surgery, intracameral mydriatics, anesthetics, mydriasis, stable pupil dilatation, Mydrane

## Abstract

***Purpose:*** To evaluate the effect of Mydrane (contains tropicamide, phenylephrine hydrochloride, and lidocaine hydrochloride) on time needed to induce mydriasis and mydriasis stability during cataract surgery.

***Methods:*** This was an observational, non-interventional, multicenter study of patients undergoing cataract surgery who received Mydrane for mydriasis and intraocular anesthesia. The study was conducted at seven ophthalmology departments at university hospitals in Poland. Patients admitted for cataract surgery within a 2-week period were asked to participate in the study. Patients whose pupils dilated to a diameter ≥6 mm after topical mydriatic administration during preoperative examinations were scheduled to receive Mydrane and included in the registry. No additional inclusion criteria were used. Patients' medical histories, examination results, and operative details were recorded. Pupil diameter was measured during surgery. Surgeons were asked to complete a Likert-based survey in parallel.

***Results:*** A total of 307 patients were enrolled. The mean pupil diameter was 7.0 ± 1.0 mm before capsulorhexis and 6.9 ± 1.2 mm before lens implementation. A pupil diameter ≥6 mm was achieved in 91.9% and 87.6% of patients before capsulorhexis and lens implantation, respectively. We asked 58 surgeons whether they agreed with the statement “Mydriasis was obtained in a short time after the administration of Mydrane”; the surgeons agreed with this statement after 92.2% (283/307) of surgeries. In addition, after 88.2% of surgeries, the surgeons agreed with the statement “Mydriasis was stable after the administration of Mydrane.”

***Conclusions:*** Mydriasis was rapidly and stably obtained after Mydrane injection, as demonstrated by pupil diameter measurements during surgery and surgeons' feedback.

## Introduction

Adequate and stable pupil dilatation is a key element of cataract surgery. Thus far, topical mydriatic eye drops or inserts have served as the standard method for achieving pupil dilatation during cataract surgery. Behndig et al.^1^ described the advantages and limitations of these methods and summarized many of the approaches used for achieving pupil dilation that have been studied during the past 30 years.^2^ We have observed increases in the use of intracameral mydriatics in contemporary ophthalmology.^3–5^ In 2015, the first ready-to-use, standardized, commercially manufactured medicinal product Mydrane (Laboratoires Thea, France) was approved for use in several European countries, including Poland.^6^ The product is injected into the anterior chamber at the beginning of cataract surgery to induce mydriasis and intraocular anesthesia. One 0.2-mL dose contains 0.04 mg of tropicamide, 0.62 mg of phenylephrine hydrochloride, and 2 mg of lidocaine hydrochloride. Mydrane is a salt- and pH-balanced solution.

Data from a phase III clinical trial have shown that Mydrane:

- induces stable mydriasis within a very short time and has a good safety profile,
- allows for reductions in doses of active substances compared with eye drops,
- speeds up surgery preparation because it is administered once at the beginning of surgery and does not need to be administered during the preoperative period.^7^

This study was a well-controlled trial whose rigorous inclusion and exclusion criteria may have prevented the results from being generalized to the entire population of patients undergoing cataract surgery. Our observational, investigator-initiated trial involved patients receiving Mydrane under conditions to which actual populations of patients undergoing cataract surgery are subjected. To our knowledge, this was the first observational, multicenter study to assess this ready-to-use product. A new study from a single center assessed pupil diameter during surgery and visual acuity postoperatively; however, this study utilized different measurement points and assessed outcomes based on patient feedback rather than surgeon feedback.^8^

## Methods

### Study design

This was an observational, non-interventional, multicenter registry of patients undergoing cataract surgery who received Mydrane to induce mydriasis and intraocular anesthesia during surgery. According to the definition given in the “Registries for Evaluating Patient Outcomes: A User's Guide. Third Edition,” “a patient registry is an organized system that uses observational study methods to collect uniform data (clinical and other) to evaluate specified outcomes for a population defined by a particular disease, condition, or exposure, and that serves one or more predetermined scientific, clinical, or policy purposes.”^9^

Surgeon opinions were collected in parallel. The operating surgeon's opinions on the times required to induce mydriasis and achieve mydriasis stability were measured on a 5-point Likert scale immediately after the end of the surgery.^10^

The study was designed, performed, and reported in accordance with the ethical principles discussed in the Declaration of Helsinki and the Strengthening the Reporting of Observational Studies in Epidemiology guidelines.^11^ The study was an Investigator Initiated Trial approved by the Ethics Committee of Warsaw Medical University (Warsaw, Poland), number AKBE/17/2017.

To ensure that our data were of the best quality, we allowed the study to be monitored by an independent local Clinical Research Organization (CRO) with experience in the conduction and monitoring of clinical trials (4 Medicine Rek PLL, Warsaw, Poland), according to the decision of heads of the ophthalmology departments leading the research.

### Patient selection

All patients admitted for cataract surgery that was performed by surgeons affiliated with the seven ophthalmology departments participating in the study within a 2-week period in March 2017 were informed about our research. Those who consented to participate in the study were included herein. The patient selection procedure was not biased, because the lists of patients who underwent surgery during the study period were created before the decision regarding the performance of the study was made, in accordance with organizational policies regarding the performance of cataract surgeries in public hospitals in Poland.

According to the Summary of Product Characteristics, “Mydrane should only be used in patients who have demonstrated, at a previous visit, a satisfactory pupil dilation with topical mydriatic therapy.”^12^ While designing the study, the investigators agreed that sufficient pupil dilation during the preoperative visit would be defined as pupil dilation to a diameter ≥6 mm, as measured as described in the protocol. The 6-mm cut-off value was adopted because the Lens Opacities Classification System III (LOCS III) scale, which is used to assess cataract maturity, requires that the pupils are dilated to a diameter ≥6 mm.^13^ Therefore, patients in whom pupil dilation to a diameter ≥6 mm (1 mm smaller during preoperative assessment than in Labetoulle's study) was achieved during the preoperative visit were eligible to receive Mydrane. The LOCS III was used with the consent of Chylack, Inc. (Durham, NC).

### Patient evaluation

Data were collected with an electronic case report form (CRF) with the MedPhantom system, which was provided by 4 Medicine Rek PLL (Warsaw, Poland). Only data pertaining to the eyes that were operated on during the study were collected. During the first visit (visit 1), demographic data and data regarding eye diseases, previous eye surgeries (including laser therapy), general diseases, and current ophthalmic and general medications were recorded. All patients underwent a full preoperative assessment (corrected distance and near visual acuity assessment, slit-lamp evaluation, dilated fundoscopic examination, biometry, Ultrasound (USG), and corneal endothelial cell count, according to the routine practices of each hospital). As iris color may influence mydriasis, iris color was assessed according to the simplified anthropological Martin scale.^14^

The pupil diameter assessment that was completed during the preoperative visit was performed with a device used for pupil diameter measurements, according to the instructions provided by the manufacturer. The instruments used in this study included a Volk Eye Check with a grid scale (Volk Optical, Inc., Mentor, OH), a Pupilometer Neuroptics (Neuroptics, Irvine, CA), and a KR-1W Wavefront Analyzer (Topcon Medical Systems, Inc., Oakland, NJ). During the first visit, pupil diameter was measured before and 20–30 min after the administration of mydriatic eye drops (1.0% tropicamide and 10.0% phenylephrine, one drop of each product, once; in Poland, only 10% phenylephrine is available). The induction of mydriasis during the preoperative visit is a routine activity and is performed in every patient because of the necessity of assessing the posterior segment of the eye.

Cataract severity was assessed according to the LOCS III guidelines. The following four parameters were graded: nuclear opalescence (NO), nuclear color, cortical cataracts, and posterior subcapsular cataracts. The use of standard medications as part of the preparation for cataract surgery at a given site (e.g., antibiotics and non-steroidal anti-inflammatory drugs) was recorded in the CRF. Patients eligible to receive Mydrane could not receive mydriatic eye drops before surgery. However, the subjects were not prohibited from receiving concomitant therapy or additional drugs. All therapeutic decisions were made by the treating physician.

Pupil diameter was measured during cataract surgery (visit 2) by using a medical ruler. Pupil size measurement (with 0.5 mm accuracy at least determined by precision of the used/available measurement instrument) was done with a medical ruler made of stainless steel (sterilizable).

Each additional drug or medical device used to induce or maintain mydriasis was recorded in the CRF. Different types of anesthesia (topical, periorbital, general anesthesia) are used during cataract surgeries in everyday clinical practice. Topical and intravenous anesthetics and sedatives can influence mydriasis. In the phase III Mydrane study mentioned earlier, the patients received topical anesthesia twice before surgery.^7^ Mydrane is an intracameral combination of mydriatics and anesthetics that is used for cataract surgery. Mydrane is a new product and is primarily considered a mydriatic agent. As this was an observational trial, we did not standardize how the patients were anesthetized. All anesthetics administered during surgery, whether by the surgeon or the anesthetist, were reported.

### Surgical technique

The surgeons used standard surgical techniques to perform cataract surgery. Ophthalmic viscosurgical devices were used in all departments, according to standard procedures. The use of trypan blue, acetylcholine chloride, or corticosteroids was noted, and all complications or adverse reactions were reported by the surgeon just after the end of each surgery (if applicable).

### Endpoints and assessments

The purpose of this study was to evaluate the effect of Mydrane on the time needed to achieve mydriasis and mydriasis stability during cataract surgery. Pupil diameter was measured at the following four key points during cataract surgery: just before the start of surgery, just before capsulorhexis, just before intraocular lens implantation, and just before the end of surgery.

Immediately after the end of surgery, the operating surgeon's opinions on the time to obtain mydriasis and mydriasis stability were assessed on a 5-point Likert scale Each surgeon was asked to rate the following two statements on a 5-point scale (strongly agree, somewhat agree, neither agree nor disagree, somewhat disagree, strongly disagree): “Mydriasis was obtained in a short time after the administration of Mydrane” and “Mydriasis was stable after the administration of Mydrane.”

### Sample size and statistical methods

The calculated sample size for a population of 278,000 (the estimated number of cataract surgeries performed in 1 year in Poland) is 246. This sample size allows us to assume that 80% of patients will have pupil dilation higher or equal to 6 mm, which is in line with expert knowledge and previous studies. The collected data were analyzed with descriptive statistical methods by using Microsoft Excel 2013. Data were expressed as the means and standard deviations, medians with ranges, or percentages where appropriate.

## Results

### Patient flow and baseline characteristics

During 2 weeks 387 patients, who consented to participate in the study, underwent a full preoperative assessment and cataract surgery. Pupil dilation to a diameter ≥6 mm was achieved in 323 patients during the preoperative visit. This group of patients was eligible to receive Mydrane. Sixteen patients received mydriatic eye drops before surgery, as decided by their surgeons, who were unable to examine the patients previously and thus decided to dilate their pupils to examine them just before surgery. These patients were not included in the “Registry,” nor were 64 patients whose pupils were dilated to a diameter <6 mm before surgery. At the time of the study, the payer in Poland did not pay for bilateral surgeries, so we did not have any bilateral operations. Ultimately, 307 patients at 7 study sites underwent cataract surgery after Mydrane administration and were included in the “Registry.” Thus, 83.5% of patients experienced satisfactory pupil dilatation (defined as ≥6 mm) during preoperative examination.

### Demographics

The majority of patients who underwent surgery were women (71.7%, *n* = 220), and the mean age of the total population was 73.8 ± 8.7 years. The median age was 74 years (range, 40–99 years).

### Ocular history

Only data pertaining to the eye that was treated were collected in this study. Based on their medical histories, 29.0% of patients had concomitant eye diseases. The most common disease was glaucoma, followed by other common types of eye diseases, age-related macular degeneration (AMD), and diabetic retinopathy. A history of eye diseases was reported by 8.5% of patients, whereas histories of eye surgery and laser therapy were reported by 4.0% and 4.0% of patients, respectively. A detailed analysis showed that some patients reported more than one eye disease. The demographics and baseline characteristics of the patients enrolled in the study are summarized in Table 1.

**Table 1.** Ocular History and Anterior Segment Findings

Concomitant eye diseases^*^	N	% out of* n = *307
Per patient		
No	218	71.0
Yes	89	29.0
Disease		
Glaucoma	41	13.4
Dry AMD	13	4.2
Wet AMD	6	2.0
Diabetic retinopathy	4	1.3
Other	36	11.7
History of eye diseases^**^	N	*% out of* n = *307*
Per patient		
No	281	91.5
Yes	26	8.5
Disease		
Corneal disease	11	3.6
Uveitis	2	0.7
Retinal detachment	1	0.3
Other	14	4.6
*History of eye surgeries*	N	*% out of* n = *307*
Per patient		
No	295	96.0
Yes	12	4.0
Surgery		
Glaucoma surgery	1	0.3
Strabismus	3	1.0
Vitrectomy	1	0.3
Other	7	2.3
*History of laser surgery*	N	*% out of* n* = 307*
Per patient		
No	295	96.0
Yes	12	4.0
Laser surgery		
Anterior segment	8	2.6
Retinal photocoagulation	4	1.3
*Conditions identified during anterior segment assessment*	N	*% out of* n* = 307*
Pseudoexfoliation syndrome	26	8.5
Iris atrophy	3	1.0
Iridotomy(-ies)	8	2.6
Iris neovascularization	1	0.3
Posterior synechiae	3	1.0

AMD, age-related macular degeneration.

### Iris color

Iris color in the treated eye was assessed according to the simplified anthropological Martin scale.^15^ The eye was described as dark (in patients with black-brown, dark-brown, and brown irises), transient and mixed (in patients with light-brown, greenish-brown, light-green, dark-gray, and gray irises), and light (in patients with light-gray, gray-blue, blue, and light-blue irises). Light eyes were noted in 49.2% (*n* = 151) of patients included in the registry, transient and mixed eyes were noted in 36.8% (*n* = 113) of patients, and dark eyes were noted in 14.0% (*n* = 43) of patients included in the registry.

### Ocular findings (anterior segment)

Specific anterior segment abnormalities were noted in the study questionnaires. The most common abnormality was pseudoexfoliation syndrome (PEX), which occurred in 8.5% patients, followed by iridotomies (2.6%), posterior synechiae (1.0%), iris atrophy (1.0%), and iris neovascularization (0.3%) (Table 1).

### LOCS III grading

For each item, a higher grade was indicative of more severe disease. Of the 307 patients included in the study, 79.2% (*n* = 243) had nuclear opacities ≥LOCS NO3.

### General diseases

At least one general disease was noted in 92.8% of patients. Most patients had more than one general disease, and a total of 587 diseases were reported by the study population. The most common general disease was arterial hypertension, which affected 78.5% (*n* = 241) of patients, followed by other chronic diseases, which affected 74.3% (*n* = 228) of patients; diabetes, which affected 23.5% (*n* = 72) of patients; prostate disease, which affected 9.4% of the total study population and 33.0% (*n* = 29) of male patients; and depression, which affected 5.5% (*n* = 17) of patients. Thirty percent of patients with diabetes were treated with insulin.

### Anesthesia

Mydrane was supplemented with only anesthetic eye drops in 86% (*n* = 264) of cataract surgeries. Additional topical anesthetics were used in 7.8% (*n* = 24) of surgeries. Periorbital anesthesia was applied in 6.5% (*n* = 20) of surgeries, and general anesthesia was used in 0.7% (*n* = 2) of surgeries. Drugs were administered by the anesthetist in 78% of surgeries. The most frequently used drug was midazolam [67.4% (*n* = 207)], followed by fentanyl [59.9% (*n* = 184)], lignocaine [21.8% (*n* = 67)], and other drugs [10.1% (*n* = 31)].

### Outcomes

The cataract surgeries for the aforementioned 307 patients were performed by 58 surgeons.

The mean and median pupil diameters in the entire study population were:

- 2.3 ± 1.1 mm and 2.0 mm (range, 1.0–8 mm), respectively, just before the start of surgery,
- 7.0 ± 1.0 mm and 7.0 mm (range, 4.0–9.5 mm), respectively, just before capsulorhexis,
- 6.9 ± 1.2 mm and 7.0 mm (range, 1.0–10 mm), respectively, just before intraocular lens implantation,
- 6.6 ± 1.4 mm and 7.0 mm (range, 2.5–11 mm), respectively, just before the end of surgery.

In *post hoc* analysis, we used a pupil diameter of 6 mm as a cut-off point to assess some secondary efficacy parameters (the percentages of patients in whom a pupil diameter ≥6 mm was achieved at defined time points during surgery). A pupil diameter ≥6 mm was achieved in 91.9% (*n* = 282) of patients just before capsulorhexis, 87.6% (*n* = 269) of patients just before lens implantation, and 75.2% (*n* = 231) of patients just before the end of surgery.

In 88.0% of cases, cataract surgery was performed, and mydriasis was achieved without the use of any additional drugs or medical devices. In the remaining 12.0% cases, epinephrine injection was the method most commonly used to achieve mydriasis. Epinephrine was used to maintain mydriasis in <0.5% surgeries. Mechanical expanders were used twice in the study population (1 Malyugin ring and 1 spatula). Additional analyses were performed in the following two groups: patients in whom no additional drugs or medical devices were used to achieve or maintain mydriasis during cataract surgery and patients in whom additional drugs or medical devices were used to achieve or maintain mydriasis during cataract surgery. In the group of patients in whom no additional drugs or medical devices were used, the mean and median pupil diameters just before capsulorhexis were 7.1 ± 0.96 mm and 7 mm (range, 4.0–9.5 mm), respectively (Table 2).

**Table 2.** Mean and Median Pupil Diameters in the Subgroups

	Mean [mm]	SD [mm]	Median [mm]	Range [mm]	> −6 mm [%]
The mean and median pupil diameters in the group of patients in whom no additional drugs or medical devices were used					
Before the start	2.33	1.1	2.0	1.0–8.0	—
Before capsulorexis	7.1	0.96	7.0	4.0–9.5	95.2
Before intraocular lens implantation	7.05	1.13	7.0	1.0–10.0	91.4
Before the end of surgery	6.72	1.31	7.0	7.0–11.0	79.0
The mean and median pupil diameters were in the group of patients in whom additional drugs or medical devices were used					
Before the start	2.14	1.01	2.0	1.0–6.0	—
Before capsulorexis	6.11	1.16	6.0	4.5–9.0	66.7
Before intraocular lens implantation	6.15	1.17	6.0	3.5–8.0	63.9
Before the end of surgery	5.91	1.44	6.0	2.5–8.0	52.8

SD, standard deviation.

A pupil diameter ≥6 mm was achieved in 95.2% of patients just before capsulorhexis, in 91.4% of patients just before lens implantation, and in 79.0% of patients just before the end of surgery.

In the group of patients in whom additional drugs or medical devices were used, the mean and median pupil diameters just before capsulorhexis were 6.11 ± 1.16 mm and 6 mm (range, 4.5–9.0 mm), respectively (Table 2).

A pupil diameter ≥6 mm was achieved in 66.7% of patients just before capsulorhexis, 63.9% of patients just before lens implantation, and 52.8% of patients just before the end of surgery.

**FIG. 1.**
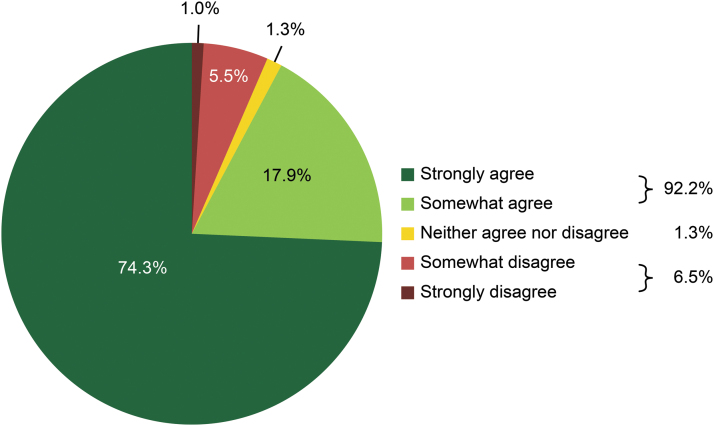
Surgeons' opinions regarding the time needed to achieve mydriasis after Mydrane administration.

We asked the 58 surgeons whether they agreed with the statement “Mydriasis was obtained in a short time after the administration of Mydrane” and found that they agreed with this statement after 92.2% (283/307) of surgeries (strongly agreed, 74.3%; somewhat agreed, 17.9%). The surgeons disagreed with this statement after 6.5% of surgeries (somewhat disagreed, 5.5%; strongly disagreed, 1.0%) and had no opinion regarding this statement (neither agreed nor disagreed) after 1.3% of surgeries (Fig. 1).

**FIG. 2.**
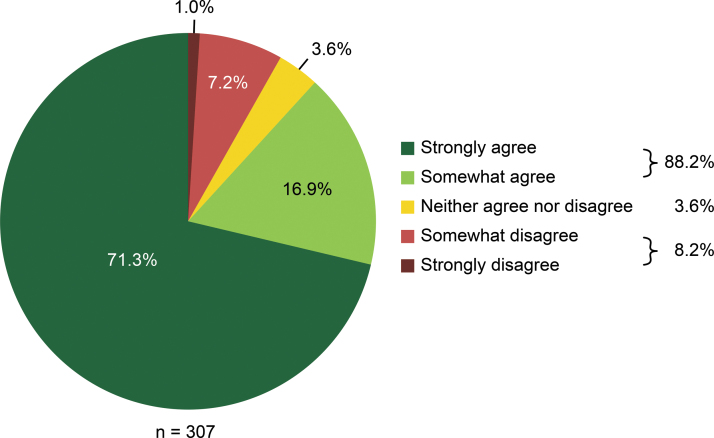
Surgeons' opinions regarding mydriasis stability.

We found that after 88.2% of surgeries, the surgeons agreed with the statement “Mydriasis was stable after the administration of Mydrane” (strongly agreed, 71.3%; somewhat agreed, 16.9%). However, the surgeons disagreed with this statement after 8.2% of surgeries (somewhat disagreed, 7.2%; strongly disagreed, 1.0%) and had no opinion regarding this statement (neither agreed nor disagreed) after 3.6% of surgeries (Fig. 2).

Only one adverse drug reaction occurred during the surgeries. Sudden miosis occurred in one patient at the beginning of surgery, just after the injection of Mydrane, and receded spontaneously. Intraoperative complications occurred in 3.5% (*n* = 11) of patients. The most frequent complication was a posterior capsule tear. Anterior vitrectomy was performed in 0.6% (*n* = 2) of patients.

## Discussion

Surgeons must obtain a very good postoperative result in the majority of patients who undergo contemporary cataract surgery. An increased emphasis has been placed on using local anesthesia and administering drugs intracamerally, and studies have shown that intracameral drug administration induces rapid and safe mydriasis.^2,7,8^ Studies have focused not only on pupil dilators but also on anti-inflammatory agents and means of maintaining mydriasis with medications injected intracamerally.^16^ The introduction and registration of the first ready-to-use combined mydriatic and anesthetic, which can be intracamerally injected, have provided surgeons with a new tool. A randomized controlled trial has shown that Mydrane causes pupil enlargement and anesthetic effects. However, each of the aforementioned studies used strict inclusion and exclusion criteria. Thus, a Polish research group was created to study the effects of Mydrane during cataract surgeries in a non-selected patient population. Patient enrolment in this study was facilitated by the use of the so-called “queue list” of a public payer in Poland. An explanation of the logistics of cataract surgeries in Poland exceeds the framework of this publication. However, it is important to emphasize that all of these factors influence patient flow (especially admission to the hospital and cancellation of surgery).

The surgeon's decision regarding how the pupil is dilated may be determined by many factors (e.g., clinical trial results, drug accessibility, and price). The most important factors are surgeon experience and surgeon opinions about products. Thus, we decided to ask each surgeon's opinion on the time needed to achieve mydriasis and mydriasis stability. To minimize bias, we asked the operating physicians to fill out the forms immediately after the procedure, as any emotions they experienced about the case would not have subsided at that point in time.

Our study had some limitations. The study was an open-label, non-randomized investigation without a control group. In addition, during the surgeries in which additional medicines were used to achieve mydriasis, pupil diameter was not measured before the injection of epinephrine. Thus, pupil dilation may have been achieved by the injection of both drugs (Mydrane and epinephrine) in these cases. Due to the observational nature of the study, it was necessary to limit the amount of data collected. Therefore, no data were collected on the type of viscoelastic, surgical settings, and the time savings. The length requirements do not allow us to present in this article the results of all the analyses we conducted. Consequently, we presented the results concerning only the primary and secondary objectives of the study.

The study also had many advantages. It was conducted at seven university hospitals. In addition, we collected data that reflected many surgeons' opinions; in particular, a total of 58 operating surgeons who performed 307 surgeries participated in this study. The study was conducted in academic hospitals. Among the operating doctors were both doctors with extensive surgical experience and less experienced doctors. According to the Polish law, a non-interventional study must not affect standard clinical practice. In the CRFs, we did not collect information about the operating doctors and their surgical experience. That approach ensured anonymity. At the same time, the sample size of the sample allowed us to generalize the results to the entire population.

We investigated the use of Mydrane under conditions to which actual populations of patients undergoing cataract surgery, who, in this study, were chosen without the use of inclusion or exclusion criteria, are subjected. Real-life studies have high generalizability, because in contrast to randomized controlled trials, they provide data on real-life situations rather than a specific set of patients who were selected in accordance with strict and controlled criteria.^17^

Almost all the patients enrolled in the study had at least one concomitant general disease, and almost one-third had concomitant eye diseases. Patients with diabetes, PEX, and prostate diseases treated with α-blockers and other drugs that increase the risk of intraoperative floppy-iris syndrome^14,18,19^ were included in the study. It is worth noting that our study population included patients with severe cataracts, because more than 45% of the patients enrolled in the study had NO, which corresponds to an LOCS III grade ≥NO4.

We also assessed secondary efficacy parameters by determining the percentage of patients in whom a pupil diameter ≥6 mm was achieved at defined time points during surgery. A study by Donnenfeld et al.,^16^ which was published in the May 2017 issue of Journal of Cataract & Refractive Surgery (JCRS), stated that the U.S. Food and Drug Administration defines intraoperative miosis as a pupil diameter smaller than 6.0 mm at any time during surgery. Thus, our assumption was consistent with the guidelines of the U.S. Food and Drug Administration. It is worth emphasizing that such miosis was reported in only 8.1% (*n* = 25) of patients before capsulorhexis and in 12.4% (*n* = 38) of patients before lens implantation. Mydriasis was achieved after a single injection of Mydrane without any preoperative mydriasis in a real-life patient population. Acetylcholine chloride was used to induce miosis in 6% (*n* = 19) of patients at the end of surgery.

Surgeons consider stable dilatation the most important factor in cataract surgery.^1^ There are no standardized questionnaires to assess the surgeons' opinion on the speed of pupil dilatation and on the stability of mydriasis during surgery. Our study is innovative in this respect. To collect surgeons' opinions, we used a Likert scale questionnaire, which is used for used for measuring opinions and attitudes, because we need a short, reliable tool that will not interfere with the work in the operating theater. Asking the doctors two questions, directly in the operating room just after the surgery, allowed us to eliminate the perceptual error. In almost 90% of cases, the surgeons confirmed that the mydriasis obtained by using Mydrane was fast and stable.

A mean pupil diameter of 7.0 mm immediately before capsulorhexis was achieved in the population of patients with pupils assumed to be satisfactory during a preoperative examination; in our study, the threshold for assuming pupils to be satisfactorily dilated during the preoperative examination was 6 mm (1 mm smaller than that reported in the aforementioned randomized study).

In conclusion, the study showed that the mydriasis obtained with an intracameral fixed, ready-to-use combination of tropicamide, phenylephrine, and lidocaine (Mydrane) during cataract surgery in a non-selected patient population is fast and stable. Therefore, the results of the study may be applicable to a broad group of patients undergoing cataract surgery. The results are based on both pupil diameter measurements performed during cataract surgery and surgeons' opinions.
